# Neither private nor new: unpacking narratives of ‘ocean privatisation’

**DOI:** 10.1007/s40152-025-00416-1

**Published:** 2025-03-17

**Authors:** Carlo Ceglia, Kimberley Peters, Philip Steinberg

**Affiliations:** 1 Department of Geography, Durham University, South Road, Durham, DH1 3LE England; 2 Helmholtz Institute for Functional Marine Biodiversity (HIFMB), University of Oldenburg, Im Technologiepark 5, 26129 Oldenburg, Germany

**Keywords:** Privatisation, Capitalism, History, Oceans, Complexity, Presentism

## Abstract

Joining others who call attention to the ways in which the ocean, its spaces, and its resources are being commodified, enclosed, and extracted in ways that benefit some at the expense of others, this paper offers a synthesis and review, echoing and extending the cautions being posited around ocean privatisation discourses and their tendencies toward simplistic conceptualisations and presentist thinking that all too often limit critical analysis. Therefore, this paper synthesises and analyses existing literature on the institutions and processes through which the ‘privatisation’ of the ocean has been, and is being, implemented, leading to two important points. First, it is showed how privatisation processes are often more complex than the word suggests. Privatisation is anything but ‘private’. The enclosure, appropriation, and rationalisation of space, resources, knowledge, and governance in the marine domain are occurring in institutional matrices where private actors operate in an array of relationships *with the state* (in its many, multiple guises), as well as non-governmental, and inter-governmental actors. Secondly, when viewing privatisation with a sensitivity to the array of institutions and actors involved, it is vital to recognise that what passes for a more recent capitalist tendency in the ocean realm rather continues long-standing, historical trajectories of violent extraction (which are equally complex in configuration). Expanding on these critiques, this paper turns to longstanding traditions that offer ways of thinking *beyond* privatisation and that engage the ocean not as a space of enclosure and extraction but as a space of relationality and livelihoods.

## Introduction

Among scholars and activists concerned with the changing political status of the world ocean, few activities have attracted greater attention than the establishment of a regime to facilitate the extraction of rich metal deposits (polymetallic nodules, rare earths, metal-rich muds, cobalt-rich ferromanganese crusts, and hydrosulphide deposits) from the deep seabed, beyond states’ continental shelves (Tassin Campanella 2023). The United Nations Convention on the Law of the Sea’s (UNCLOS) designation of the seabed beyond the continental shelf as ‘The Area’, its designation as ‘the common heritage of [hu]mankind’, and the empowerment of an international organisation linked to the United Nations system – the International Seabed Authority (ISA) – to manage resource extraction there all signify a move towards the seabed’s enclosure (Zalik 2018). However, *enclosure* is not necessarily the same as *privatisation*. Indeed, enclosure of the seabed is occurring at a number of scales. Globally, the ’common heritage’ designation encloses the seabed for the community of all people (and, in some interpretations, future people as well) (Borgese 1998). On a national level, the creation of the ISA, as a UN institution, facilitates enclosure by the community of states (Ashworth 2023). And within the ISA, the structure of decision-making exists on yet another – smaller – scale, with power concentrated in the secretive Legal and Technical Commission: a further act of enclosure by an elite *within* the ISA (Willaert 2020). Arguably, the juridical separation of the seabed from the water column above, which facilitates UNCLOS’ seabed regime, is another form of enclosure (Ranganathan 2019). As various scholars of contemporary seabed politics have noted, these processes have been driven by (and have facilitated) a mission to *commercialise* or *commodify* the seabed’s riches (Standing 2022). Indeed, in The Area individual corporations have asserted (and gained) power through the provision of capital and technology. However, at the same time, states have been exercising (and increasing) leverage through their position as signatories to UNCLOS, and the ISA has asserted legitimacy through its position as guardian of the ‘common heritage’. Clearly something is going on here that is more complex than the definition of privatisation as the ‘transfer of exclusive property rights […] to private actors’ (Schlüter et al. 2020:1).

Indeed, definitions of privatisation suggest a process that is essentially linear: Rights are successively transferred from the public to the private domain, and over time more and more aspects of life (and space) become privatised. However, as the case of the deep seabed illustrates, the rearrangement and exertions of power over space are complex and have longer histories. On the seabed, enclosure, appropriation, and rationalisation of space, resources, knowledge, and governance are occurring in institutional *matrices* where private actors operate in an array of relationships with state, non-governmental, and inter-governmental actors. Furthermore, this process is not recent. While deep sea mining is often regarded by both its proponents and its detractors as the final oceanic frontier (see Hartley 2012; Fritz 2015; Kirkham et al. 2020; amongst others) – a space ripe for enclosure and commodification – others have shown that the seabed has long been a frontier to capitalist exploitation and extraction (see Saputra and Sammler 2024 on the imperial drivers of nineteenth century tin seabed mining offshore of Indonesia, for example).

In the remainder of this article, we extend past the deep seabed to develop a broader critique of understandings of ‘ocean privatisation’ beyond this specific example. We do so from particular situated perspectives, and drawing from selected secondary materials, offering an article that reviews and synthesises literature pertinent to this debate. What this paper offers, then, is an overview of debates arguing for continued, and greater, attention to be paid to the complex dynamics of privatisation. Returning to our perspectives: this paper is rooted/routed in thinking *spatially* about privatisation – its location within the aforementioned institutional matrices that complicate public/private assumptions, and its place within colliding, overlapping, repeating notions of time, where the past echoes into present, the present carries the past, and futures are imagined and enacted in the violences of now. In the sections that follow we also stress this spatial approach in that the dynamics of privatisation witnessed are not oceanic, per se. Notwithstanding the special nature of the sea as a spatial site of dynamism, mobility, and liquidity (see Steinberg and Peters 2015), here we show how privatisation exceeds the ocean as a spatial phenomenon, less bounded and exceptional than boundless and excessive (see Peters and Steinberg 2019). Our focus, thus, is less on how privatisation operates *in* space, but rather its economic trajectories and political projections *through* space (and time).

Working through a diverse range of literatures — from theoretical geography to practical marine management, and from mercantile-era history to contemporary political economy, and drawing on a range of examples from around the world – we reflect on two, related ways of thinking that permeate much of the literature on the topic of privatisation. First, we focus on the narrative’s *lack of complexity*: even as private actors influence the shape of ocean space, and at times lay claim to elements of it, these actors are always entangled with, and sometimes acting at the behest of, state actors. Privatisation, we argue, is rarely, if ever, strictly ‘private’. Secondly, we focus on the narrative’s *presentism*: all too often the innovations of present institutions are viewed apart from the context that generates their emergence and to which they often owe a historical debt. Thus, our second argument is that privatisation is not particularly recent. In the concluding section, we consider why these perspectives on ocean privatisation matter: not simply to carefully situate these concepts spatially and temporally within academic practices in the Global North; but also, more fundamentally, to expand the dialogue to conceptualisations of governing the ocean that depart from the hegemonic norms of the capitalistic imaginary. By understanding privatisation not as a singular historical endpoint but as a continuum of possibilities, histories, and institutional arrangements, we advocate the continued necessity for reconceptualising, or undermining, it.

### Privatisation is not private

In a recent, pivotal piece on privatisation and the ocean Schlüter et al. (2020:1, emphasis added) define privatisation as a means of ‘*transfer*’ of ‘exclusive property rights over valuable goods, spaces, and processes to private actors, be they individuals, corporations, or nongovernmental entities’. Here the authors acknowledge that privatisation involves matrices of organisations to whom ‘exclusive property rights’ are granted. In other words, there is a complex enmeshment of people and institutions involved in such processes. Spatially, this definition is not bounded to oceanic instances of privatising dynamics but accurately describes most instances of privatisation on land, when the state typically relinquishes its authority to build or manage a piece of infrastructure, or to deliver a social service, contracting state rights to the highest bidder. For example, literature on agrarian reform and change deals deftly with the politics of privatisation (see, for example, Fraser 2008; Green 2022). However, in the ocean the transfer of rights to a private entity (whether individual, corporate, or NGO) is more often than not accompanied by a degree of state enclosure rather than state abandonment (see Havice 2021). Mallin and Barbesgaard’s (2020:121) assertion regarding the logics driving the ‘blue economy paradigm’ – that the ‘enthusiasm’ for blue growth involves ‘enveloping supranational institutions, state governments and development financiers’ – applies as well to the broader universe of ocean governance, conservation, and development initiatives. Indeed, what is visible in literature on processes of privatisation at sea – from fisheries management to maritime security, to name just a few (see Fawcett et al. 2022; Gould 2021; Hadjimichael 2022; Hannesson 2004; Havice 2021; Mallin and Barbesgaard 2020; Weir and Kerr 2019) – is that privatisation is often a process by which the state *gains* control and *asserts* power, just as is true more generally in the neoliberal economy on land (Harvey 2005).

To develop this point further, and to simultaneously voice the import of understanding privatisation as ‘anything but private’ in its interweaving with the state, we first turn to Becky Mansfield’s pivotal work on the establishment and development of the Alaska pollock fishery – one of the most abundant single-species fisheries in the world – in the North Pacific Ocean off the coast of the United States (Mansfield 2001, 2004a, b, 2007), before drawing out an example from Carlo Ceglia’s research in the Republic of Seychelles and, finally, turning to ocean infrastructure examples derived from the work of Laleh Khalili’ (2021) and, briefly, Starosielski (2015). Although necessarily selective, the examples in this and the next section have carefully been chosen to work across a diverse range of disciplines to demonstrate the extent to which our propositions spatially and temporally reverberate within multiple geographies, socio-political histories, and oceanic realms.

In 1976, amid the simmering climate of the Cold War and the political turmoil of the decolonisation waves, the United States government officially extended its jurisdiction over fishery resources to a distance of 200 nautical miles from its coasts (Mansfield 2001). One of the stated aims of the policy was to ‘Americanize’ the pollock fishery – that is, to create a strong domestic industry, which was virtually non-existent at the time – both at sea and on land and consequently crowd out foreign interests (and vessels) from what was then considered a US resource (Mansfield 2004b). Although heavily dependent on foreign direct investment to maintain and expand land-based processing infrastructures (which were mainly foreign-owned), the state’s efforts to establish a domestic fishing capacity for pollock could be considered successful by the 1990s – so much so that the previous crisis of ‘underutilisation’ was now rescripted as one of ‘overfishing’ due to a perceived surplus of fishing capacity (Mansfield 2004a). This led the US government to embark on a campaign to reduce capacity in that fishery through privatisation via the 1998 American Fisheries Act. The Act enacted an extensive series of privatisation reforms including closing the fishery to new entrants, instituting total allowable catch (TAC) thresholds, and dividing the fishers into cooperatives with annual quotas. Subsequently, shares in cooperatives were leased, which led to further concentration of the fishery in private hands.

Nonetheless, although often characterised (and promoted) as a market-based approach to face both the ecological and economic crisis looming over the fishery, these regulations were the result of intense negotiations prompted by specific local politics and ecological histories alongside a state preoccupation with ‘Americanization’. The ‘privatisation’ of the pollock fishery was hence anything but a retrenchment of state power from the industry and the rise of unfettered market forces (Mansfield 2004b). As Mansfield details, the elevation of market forces in the pollock fishery was but one component of a broader, *state-led agenda* to manage competition and investment across a range of fisheries and, ultimately, to structure fishers’ investment strategies and livelihoods. In short, as Mansfield (2004b:574) notes, ‘to protect competition, the Act place[d] limits on the extent to which market mechanisms influence activity in these industries’. For our purposes here, the history of the pollock fishery in the US North Pacific Ocean, from its inception to the present day, reveals the manifold tensions – political, economic, and social – that translated into a set of regulatory measures towards a privatised fishery through neoliberal modalities of state enclosure and control, a process that speaks as much to increased *state* control as it does to unfettered privatisation^1^.

Just as privatisation shows enhanced state management when one focuses on a specific fishery (e.g., the North Pacific pollock fishery), much the same can be said with reference to more wide-ranging ocean privatisation programmes. Indeed, recent interventions in the governance of ocean spaces under the banner of the ‘Blue Economy’ offer further analytical insights into emergent (but mutually supportive) relations between the private sector and state(-making) practices. Through a loosely integrated series of initiatives designed to attract international financial capital and political will, ‘Blue Economy’ projects have been mobilised to spur ocean-based economic growth, environmental stewardship, and social development (Gruby and Campbell 2013; Hadjimichael 2018; Silver et al. 2015; Voyer et al. 2018). An example of such an initiative is the one jointly sponsored by the Republic of Seychelles, an archipelagic state in the Western Indian Ocean, and The Nature Conservancy (TNC), one of the largest environmental NGOs in the world^2^.

Navigating the devasting 2008 global financial crisis that led the Seychelles government to declare bankruptcy and to embark on a series of IMF-led structural adjustment programmes, Seychelles’ state officials turned to the ocean, the space forming over 99% of their territory, as an avenue of potential socio-economic and environmental development (Silver and Campbell 2018). Although a few years in the making before launching it on the world stage, in 2015 Seychelles and TNC officially launched a debt conversion that restructured a portion of Seychelles’ sovereign debt so as to free up, and allocate, a budgetary space to fund ocean conservation and development in the archipelago (Convergence 2017). As part of the deal, TNC was tasked with brokering responsibilities (between Seychelles and its creditors), raising funds for the debt buyback (through private and philanthropic organisations), designing a Marine Spatial Plan (MSP) for Seychelles’ 1.37 million km^2^ Exclusive Economic Zone, and providing technical assistance with the MSP implementation process. The deal received accolades for two reasons: first, it was heralded as the first-ever debt restructuring to cover ocean spaces (for instance, Carrington 2018); secondly, it put in place a mechanism to solve a series of perceived flaws that marked the initial, pioneering land-based debt restructurings of the 1980s – mainly relating to governments siphoning off restructured debt for purposes not agreed upon (Sheik 2018). In essence, the deal with TNC mandated that the government of Seychelles establish what was called a Special Purpose Vehicle in the form of a public-private trust fund to manage the financial transactions associated with the restructuring, and to allocate the freed-up debt (and any new stream of money) for ocean activities (Christiansen 2024). Specifically, the Trust has a governing structure where two *Ex-Officio* Directors, one government representative and one TNC representative, have special veto powers as per the Establishment Act of the Trust (Conservation and Climate Adaptation Trust of Seychelles Act 2015; Conservation and Climate Adaptation Trust of Seychelles (Amendment) Act 2022; also Silver and Campbell 2018).

At root, then, the Seychelles Blue Economy initiative appears to be an instance of the Seychelles government privatising its marine resources by surrendering degrees of sovereign authority to the non-governmental TNC and to private financial capital (in some cases mediated by intergovernmental institutions like the World Bank) that provide investment (Christiansen 2024; Silver and Campbell 2018; Schutter et al. 2021). And yet, after more than ten years in the making, the specifics of what is at stake for Seychelles, TNC, the Blue Economy, and their entanglement with ocean space are still far from settled. Recently conducted research in Seychelles points in that direction. For instance, officials and key actors in the Blue Economy project in the archipelago are acutely aware of the uncomfortable position they entered through the deal – but as Seychelles’ former ambassador to the UN, Ronny Jumeau, recounted, when people approach him worried about the ‘agenda’ TNC has that might go counter to Seychelles’ interests, he replies, ‘Yes, TNC has an agenda, but so do we’ (personal communication with Author 1, 30/11/2022). Jumeau conceded that Seychelles has indeed ceded part of its sovereign power over its ocean space, but he quickly pointed out that this is a *temporary and partial* concession willingly agreed to by the government in light of the substantial benefits it brings (long-term financial flows, international visibility, capacity building, capital investment, among others). In their quest to diversify an economy heavily reliant on fisheries and tourism, while struggling with the limitation of a ‘small island state’ (e.g., limited human and technical capacity), Seychelles’ state officials are leveraging their ocean territory while actively enrolling the technical know-how of international institutions to advance their own visions of ocean development in the current geo-political climates. Although the process is not without contestations (Christiansen 2024; Kılıç 2024), with such a move Seychelles is trying to assert independence through ‘reckoning interdependencies well’ (Clifford 2001:474; see also Steinberg and Chapman 2009). In other words, far from being a simple ‘transfer of exclusive property rights […] to private actors’ (Schlüter et al. 2020), the privatisation modalities – understood as the opening up of ocean governance and economy to private actors, be they local or international – enacted in the Seychelles’ case reveals an ocean-sovereign space that is malleable (Duffy 2006), where the Seychelles’ state is actively reimagining its own future vis-à-vis local geomaterial affordances and transnational pursuits, which include, but are hardly defined by, increased cooperation with private actors (see also Bueger and Wivel 2018; Saddington 2023).

While the examples of the US North Pacific pollock fishery and Seychelles’ Blue Economy project each speaks to ways that state-led marine privatisation initiatives in fact reflect a continued (and perhaps increased) entanglement with state power, this phenomenon can also be observed when one turns to global marine infrastructure projects – helping to further reiterate the complex matrices of relations that challenge any straightforward assumptions about privatisation at sea. For instance, Laleh Khalili’s (2021) analysis of maritime logistics infrastructure and Nicole Starosielski’s (2015) account of the telecommunications industry within marine spaces both explore the precarious and contested flash points where multiple colonial pasts, postcolonial geopolitics, lively ecologies, and social relations make certain spaces productive for capitalist development. In doing so, each of their analyses complicates singular notions of privatisation. Each author documents the infrastructural lives of a crucial, albeit often invisible, highly capitalized, mainly privately owned global industry that sustains global connectivity: shipping (Khalili 2021) and telecommunications (Starosielski 2015). Extending historical imaginaries that construct the ocean as conceptually empty (or at least emptiable) (Steinberg 2001), both industries deploy the ocean as a place to be traversed as smoothly and efficiently as possible, to allow for the friction-free circulation of goods, capital, and information (at the oceanic surface for the shipping industry, at the depths of the seabed for the cable industry).^3^ Although these mobilities are undertaken primarily by private entities, they require states – individually and as a collective state system – to develop and maintain legal and logistical infrastructures – an institutional requirement that, in the case of submarine telecommunications, goes all the way back to the development of the first Trans-Atlantic telecommunications cable in the 1800s, an endeavour that relied on a mix of state and private activities for its eventual success (Steele Gordon 2003). Furthermore, even as these infrastructures maintain the ocean as an ‘empty’ (but managed) space to move through, the ocean itself provides a material environment (Steinberg and Peters 2015) that appropriates, and can be appropriated by, state and private practices of control and surveillance (see, for example, Peters 2014).

Focussing more closely here only on Khalili’s (2021) work on one of the most prominent, global, oceanic industries today as in the past – maritime transport – to further illustrate these points, shipping in the Arabian Peninsula today continues long-standing legacies of imperial exploitation and expropriation for distant economies, where legal practices function as but one set of instruments through which such legacies are materialised. For instance, Khalili (2021) recounts how arbitration tribunals have been weaponised by transnational corporations and their respective governments to extract (economic and political) value from the region. A famous example detailed by Khalili is the 1956 case where the Saudi government was brought to an arbitration tribunal in Switzerland by the Arab American Oil Company (ARAMCO), backed by the US government, for a supposed breach of the 1933 Oil Concession Agreement that the Saudi government had signed with ARAMCO. ARAMCO believed the arbitration necessary because of the recently signed agreement between the Saudi King and a Greek shipowner, Aristotle Onassis, that conferred to Onassis the exclusive right to ship Saudi oil with his tankers under the Saudi flag – a right that US and British diplomats felt would infringe on their concessions, and hence their monopolies in the region, jeopardising the control, extraction, and distribution of the resource. Inaugurating a trend that still dominates today, the tribunal ruled in favour of ARAMCO on the grounds that the Saudi legal system did not provide any clear set of regulations on the exploitation of oil deposits and that such a lack was to be filled in by the ARAMCO Concession Agreement. Khalili quotes from the verdict: ‘Any Saudi claim to jurisdiction over its own borders or maritime business was ‘contrary to the needs of international commerce and involved a restriction of the principle of the freedom of the high seas unjustified under international law’. In other words, the high seas had to be ‘free’ for Aramco to do its business – though not for Saudi Arabia’ (Khalili 2021:71). Although the ultimate resolution in this instance may be seen as privatisation (the transfer of resources from Saudi Arabia to ARAMCO), it was achieved through a construction of ocean space that drew upon various levels of formal and informal legal, political, and military powers exercised by *states* and institutions of the *state-led* international system.

Furthermore, Khalili (2021) shows how legal precepts and institutions can also directly draw on notions of ‘ocean privatisation’ to generate new configurations of sovereignty at sea. In the Arabian Peninsula, as around the world, sovereign states are enrolling international commercial legal practices to create so-called ‘free zones’ at ports, arguably to boost maritime commerce and capital attractiveness. Essentially, a free zone is an area where ‘there is little or no corporate tax, little or no income tax for noncitizens, no customs or tariffs, and very little regulation. In a sense, they are offshore spaces but onshore, where legal striations allow accumulation of capital without restraints’ (Khalili 2021:83). In other words, on the coastal margins, as in the ocean’s depths, the malleability of the ocean as simultaneously subject to, constitutive of, and external to the territory of the sovereign state is actively moulded to fit neoliberal modalities of value generation for private capital and allied state actors in a manner that is neither wholly private nor wholly public.

### Privatisation is not new

The examples from the previous section – on US fisheries, Seychellois marine-based development, and Arabian ports – all illustrate the heterogeneity of ocean privatisation practices and how narratives in the mainstream imaginary may lack complexity. Returning briefly to Schlüter et al.’s touchstone piece on privatisation (2020:1, emphasis added), the authors note how it is a ‘*process*’ of ‘transfer’ of ‘exclusive property rights’. Here the word process is important in highlighting that this is a phenomenon that happens *through* time. Privatisation does not occur at a singular moment, when control shifts from one ‘state’ (in all meanings of the word) to another. As the Arabian ports example suggests, past, present and future collide in driving the dynamics of privatisation. However, narratives often stress a progressive drive toward ocean privatisation with a tendency toward presentism. That is, here in the second part of the paper, we posit (with others) that privatisation is not only not private; it is also not *new*. Again, Mallin and Barbesgaard’s analysis with respect to the specific example of the Blue Economy has broader resonance for ocean governance:

Inquiries [about the blue economy] easily skim over the long historical lineage of capitalist modes of enclosing, appropriating, carving up and commercialising the seas […] we posit that the blue economy moment may be more adequately investigated and understood as part of a *longue duré [sic]* transformation of capitalist relations with the sea. (Mallin and Barbesgaard 2020:122)

Moving away from the contemporary articulations of Blue Economy projects towards a more established ocean industry, Liam Campling and Elizabeth Havice (2014) similarly problematise recent attempts at theorising the relationship between capitalism and industrial fisheries. Empirically and analytically mapping the concept of *property* and *property relations* within the industry, they submit that ‘mainstream analyses’ stressing the tragedy of the commons narrative with their consequent, logical solution of the institutionalisation of private property rights fail to account for the intricate, multiple trajectories at play within the ‘fisheries crisis’ (Campling and Havice 2014:723). Instead, Campling and Havice

illustrate the development of property relations in the oceans, the role of (changing) powers in shaping them, and a historically, politically and ecologically contingent theory of rent [arguing that] the complex roles and multiple logics of states deserve careful conceptual and empirical attention in the narrative of the fisheries crisis. (Campling and Havice 2014:724)

In short, then, privatisation, rather than being something that *arrives* with contemporary global ocean organisation, is a phenomenon that has looped, repeated, endured, and stretched from past to present, again in multiple and complex ways (Campling and Colás 2018). Today’s complex state- and more-than-state, private, and non-governmental matrices of organisation of maritime or oceanic-based industries have historical anchorages that reverberate into the present.

Drawing from amalgamated literatures on privateering (with the very word ‘private’ underpinning its logics), alongside a further case of oceanic lines and designations, provides useful examples. Starting with the former, in the 17th and 18th centuries, privateers effectively engaged in seemingly ‘private’ acts of violence at sea (see Colás and Mabee 2011). Private persons and collectives – ‘investors’ – ‘took up the challenge’ of a ’business opportunity […] fitting out a ship, manning it and supplying the cannon balls and powder necessary to fight’, all in ’anticipation of the prize money that would be the reward of a successful cruise’ (Ogborn 2008:172). Yet they did this under the remit of the state, with privateers – privately facilitated ocean endeavours – possessing letters of marque that were issued by states with the ultimate goal of asserting sovereignty. As actors ‘waging public wars by private means’ (Heller-Roazen 2009:81), the case of privateering encapsulates our argument that ocean privatisation is neither wholly private nor particularly new. As Ogborn neatly puts it:

The organisation of maritime violence in the early modern era was a public-private partnership. The state hired or required merchant vessels to become ships of war, giving them commissions to fight the enemy and to share in the ‘prizes’ … The vital element that made legitimate this form of waging violence at sea for private profit was authorisation from a government that the privateer was acting in its name. (Ogborn 2008:173)

In other words, privateering can be best understood as a state-sanctioned activity carried out by non-state actors who were able to extract wealth for private gain *and* reproduce the notion of sovereign statehood in unbounded ocean-space. Like the examples from the previous part of this paper, privateering can be thought of as neither a wholly private nor wholly state enterprise. What may at first glance look like a devolution of state authority in many ways facilitated the state’s further accrual of power.

In searching for a more ‘pure’ form of privatisation in the 17th and 18th centuries one might be tempted to turn to piracy. Whereas privateering was carried out under state sponsorship, piracy was inter-ship crime *without* state sanction. In practice, however, the distinction between pirate and privateer was not so neat. Letters of marque were frequently forged, retracted, or granted after the fact, casting acts of privateering into doubt and blurring the line between privateer and pirate. Indeed, individuals (and boats) frequently crossed the pirate-privateer line depending on opportunities available (Benton 2010; Heller-Roazen 2009). The examples of privateering and piracy hence alert us to a centuries-old pattern of ocean privatisation existing in complex matrices that bring together public-private and state-non-state. They also point to the linkages between ocean privatisation and violence, which remains a concern today (for two very different examples, see Baker-Médard and Kroger 2024 and Dua 2019).

The recognition of links between historic and contemporary acts of ocean privatisation allows us to trace the very logics that have underscored the phenomenon, from privateering on through the planning of contemporary ‘Blue Economy’ initiatives. That the ocean can be privatised in different ways rests on the very historic *construction* of the ocean in the first place: the ways in which oceans have been imagined, produced, and practised (and by whom, with what power and authority to define the oceans in such a way) (see Steinberg 2001). Our point is: one can only privatise on the proviso that a space is constructed as ripe, or ready, for privatisation. On land, such a construction has historically relied on ‘moments’ that, following Gavin Capps’s (2016) analysis of modern landed property, are of ‘separation’ (e.g., of wage-labour from capital), of ‘appropriation’ (e.g., of the monopoly power over land and resources), and of ‘elimination’ (e.g., of legal and political barriers weakening accumulation processes). With renewed intensities today, a variegated constellations of financial, scientific and institutional configurations are further fabricating spaces as amenable to privatisation through contemporary practices of (re)valuation of natures for capital accumulation within the Green Economy paradigm – itself a precursor to its Blue counterpart – that have been variously theorised as forms of ‘green grabbing’ (e.g., Fairhead et al. 2012).

For our purposes here, the establishment of ocean routes, similarly to what would later happen to ocean zones, where allocations of space were ‘owned’ or ‘belonging’ to nations, was crucial to subsequent processes of privatisation. Although the designation of the High Seas (for the time being, at least) as beyond appropriation may at first glance appear to contradict the overarching logic of the ocean as ripe for some form of privatisation, this contrapuntal construction nonetheless supports the overall notion that ocean-space may be allocated when the conditions are ‘right’ (Steinberg 2009). Indeed, the High Seas have historically been amenable to privatisation, at least when one adopts the broad and multifaceted definition of privatisation advocated here. For example, going back to the 1490s, forms of oceanic allocation could be witnessed through Pope Alexander VI’s designation of oceanic routes (and by default, the areas of the ocean in which those routes were found) to Spain and Portugal (see Steinberg 1999). At this point, the sea was not determined as a space of a particular state, or enterprise, but something beyond the land, for which nations began to vie. As Steinberg (1999, 2001) notes, with the realisation that ‘distant’ lands could be conquered in acts of colonialism – to build prosperity and wealth – European nations quickly sought to establish routes across the space between: the sea. They were helped in doing so by exactly the kind of complex state - non-state configuration that has already been described, whereby, in the interests of the state (and in the interests of spreading Christianity), the Pope – *acting as a private agent for the state* – ‘divvied’ up the world’s ocean routes via the Papal Bull (1493) and later the Treaty of Tordesillas (1494) (see Steinberg 2001).

This example shows how private institutions and persons had a hand in claiming and extracting resources from the sea: in this case in the shape of routes. Yet it is these lines drawn in the ocean that also begin to more formally raise questions with respect to ownership and control of watery realms (and the resources within them). Indeed, in the centuries that followed the Papal delimitation of ocean routes, the status of the ocean – as a space of national territory or as a commons for all – would be debated and disputed. As Havice and Zalik (2018:219) note, ‘[d]istinct areas of the oceans and specific resources can and have been treated as open access, common heritage, public goods, state property, and/or private property’. In the 1600s, for example, Hugo Grotius’ hallmark text ‘Mare Liberum’, or the Freedom of the Seas, advocated that the sea (beyond the cannon fire range) could not be owned by sovereign states but instead should be open to use by all. This doctrine of a ‘High Seas’ beyond state possession, which even in its very origin was designed to justify the projection of Dutch naval power to the East Indies, went on to enable the ocean’s capitalist potentials to be exploited (in other words, for use by ‘private’ enterprise). However, this in itself is a state-sponsored norm that indirectly extends the exclusionary power of the state system, if not of individual states, to the ocean commons.

Grotius’ designation of a High Seas oceanic commons is reproduced in UNCLOS. Indeed, today under UNCLOS, as in previous centuries under Grotius, the High Seas regime that limits enclosure by individual states can, through enclosure by the state system, facilitate appropriation by private businesses – a dynamic that is also occurring in the ‘global commons’ of outer space, including its routes (i.e. orbits, see Beery 2016) and its resource-rich places (i.e. celestial bodies like the moon, see Klinger 2017). These systems of ‘soft’ enclosure are, like elements of privatisation, dynamic and adaptive, and today there are indications that, even in the High Seas, small steps are being made toward state enclosure through such innovations as High Seas Marine Protected Areas (Leenhardt et al. 2013) and treaty-based High Seas ocean governance initiatives like the Central Arctic Ocean Fisheries Agreement (Dodds 2019; Molenaar 2016).

Given the long history of ocean privatisation (in its various forms), we are led to ask: how does privatisation come to be understood as new (and not old)? How has the past come to be overlooked in the present, to the point where contemporary scholars seem to couch privatisation as something new or emergent? Although we cannot answer definitively, the rise of the ‘Blue Economy’ discourse may be central here. The ‘Blue Economy’ refers to a suggested shift in the intensification of (economic) use of ocean resources, but with sustainability in mind (Martínez‑Vázquez et al. 2021). Although Martínez‑Vázquez et al. (2021:1) note that the ocean has always been used for ‘economic activities’, they identify the Blue Economy as ‘a recent field of study that encompasses economic activities that depend on the sea, often associated with other economic sectors, including tourism, maritime transport, energy and fishing’ but which is ‘respectful of the environment’. Simultaneously, critics of the term and the ideological project behind it note that efforts to implement a ‘Blue Economy’ have allowed for unfettered capitalist extraction (Bennett et al. 2021) and hence call for degrowth strategies (see Hadjimichael 2022) and attention to the human rights of ocean peoples (Ertör 2023; Satizábal et al. 2024). Like its proponents, even its detractors tend to recognize the ‘Blue Economy’ as an event. The very term ‘Blue Economy’ catalyses a *contemporary* policy reality of ‘blue growth’ that is conjured up the moment it circulates as a discourse, but that still sits uncomfortably with the lived histories of the receiving communities – as the pioneering case of Seychelles demonstrates (Ceglia 2024).

The relative newness of the term (it first appeared in 2009, according to Martínez‑Vázquez et al. 2021:1) arguably works to erase past ocean privatisation by positing blue growth and the use of the oceans as ‘a *new* frontier for economic development’ (Bennett et al. 2021; emphasis added). Whilst there are a variety of ‘new’ technological industries entering the ocean realm, by understanding private interests in driving economic uses of the ocean as ‘new’ one denies a longer history behind current instances of privatisation. This presentism pits the past against the present, with the present associated with more intensive (ab)uses at sea/of the seas. But past, private, ocean uses were hardly less intensive, or violent. To highlight one particularly egregious example, the transatlantic trade in enslaved peoples witnessed private businesses (with and without state interests) make huge economic gains through a mode of oceanic economy, engaging in the forcible, violent displacement of millions of people in a maritime trading circuit that reduced humans to commodities within a system of capitalist production and accumulation (James (2001 [1938]), Rediker 2008; Sharpe 2016; Williams 1994; Yusoff 2018). In short, when one takes a long view of the ocean’s history and future, privatisation, in its various forms, is an ongoing, variegated process, not a singular moment in time.

### Why this matters: conclusions

This article reads the spatial complexity of privatisation (its configuration in webs and matrices), and the complexities of its temporalities, mainly through a synthesis of the literature – bringing together findings to further the case for thinking past dominating narratives of privatisation as a phenomenon that involves only private agents/agencies (rather than also the state), and one that is a more recent, rather than enduring, occurrence. But what is the significance of reiterating these lines of argument? In collating them, we have demonstrated with a necessary intensity and focus how it is crucial to decouple understandings of ocean privatisation from an emergent, linear narrative where rights are successively transferred from the state to private entities as the ocean economy expands into new domains. Instead, spatially and temporally locating it through this lens, we bolster privatisation as neither strictly private (i.e. an abrogation of state authority) nor fundamentally new (i.e. a unique aspect of late capitalism as it expands to ‘new frontiers’). In this final section, we explore some of the perspectives that emerge when ocean privatisation is analysed from a spatial-temporal lens better attuned to, but also free from, such thinking.

For a start, by being aware of, but also by turning away from, presentism it is possible to develop historical insights into patterns of oceanic privatisation, past and present. Ogborn (2008:44), in his extensive work regarding maritime worlds, labour, control, and capital from 1550 to 1800, shows how complex networks and flows, ‘built, extended, sustained […] through people, goods, ships’, present us with modes by which the seas and worlds accessed through them were subject to privatising forces. More recently, Campling and Colás (2018:778) have asserted: ‘[t]he sea has been a protagonist in the development of capitalism from the very beginning – capitalism is a world-system emerging out of maritime trade during the long 16th century (1450–1650)’. There is a danger that if one adopts a presentist attitude toward the privatisation of the ocean this past becomes obscured (see also Mallin and Barbesgaard 2020).

But presentism, together with less nuanced conceptualizations of privatisation, also prevents an appreciation (or even acknowledgement of, respect towards, or listening to) understandings of the ocean that provide framings beyond these limits and that are crucial for imaging futures that are not an alternative to, but an already existing way of conceiving of, living with, and relating to, the water (see, for example, George and Wiebe 2020; Lobo and Parsons 2023). As we have demonstrated, ocean privatisation (historic and contemporary, and with varying degrees of state involvement, on the High Seas and in coastal waters) has its roots in a specific Western conception of ocean-space as amenable to privatisation – in the words of George and Wiebe (2020:500), it is ‘resource’ rather than ‘force’. It also has roots in Western imaginaries, and practices, of exclusive property rights. These points are significant, because such imaginaries are powerful in that they violently crowd out other ways of knowing, or thinking about, ocean-space that stand apart from privatisation and its associated practices^4^. Indeed, the lack of historicisation of oceans creates an amnesia about the ways that, for centuries, ocean peoples have lived *with* and *continue to live with* the seas around them (Hau‘ofa 2008). As George and Wiebe (2020) note, the idea of the ocean as ‘separate’ underpins its construction as a space of extraction, but this is a historically, and culturally, specific construction of ocean space. As Fawcett et al. note,

Historically, the seas have been the site of the violence of the slave trade, world wars, and the overall bolstering of imperial and colonial structures. The prevailing knowledges from these historical processes have largely ignored Indigenous relationships to the ocean […] But what knowledges and epistemologies, Indigenous sciences and research autonomous from proprietary, extractive purposes, have been lost or gone unheard in these processes? (Fawcett et al. 2022:77)

This is not to associate Indigenous voices as history (another violent trap of how Indigenous ocean worlds – and their multiple, heterogeneous ways of knowing – are often presented as of the past, not the present or future), but rather to make the point that Ocean Peoples have long articulated – and practised – ways of living with the seas that do not have capital accumulation – and the privatisation that drives it – at its core. Indeed, in a paper that addresses decolonial *futures*, ocean citizenship, and relationalities with water worlds, George and Wiebe (2020:499) highlight epistemologies that challenge ’extractivist, neoliberal settler colonial governmentality across colonial jurisdictions and boundaries’ – acts of privatisation that construct the ocean as a resource frontier (see also Havice and Zalik 2018; Fawcett et al. 2022). George and Wiebe (2020:500–501) articulate how Indigenous ways of knowing situate water by ‘turning away from landlocked property-centric territorial geographies’. This is echoed in Hau’ofa’s longstanding work on ocean relationality (2008, see also Lobo and Parsons 2023). Fawcett et al., also drawing from George and Wiebe, note that,

George and Wiebe (2020: 4) analyse from epistemologies across archipelagos in Kanaka Maoli (Native Hawaiian) and Coast Salish (First Nations) how to “challenge the foundational underpinnings of extractivist, property- centric settler- colonial liberal governmentality by turning away from land- locked property- centric territorial geographies and engage with more embodied, fluid, storied, and vibrant ways of being, knowing and sensing the world”. The Consortium for Ocean Leadership (2020) recent workshop to identify national ocean exploration priorities in the Pacific reports the vital necessity of “sustained interactions with Indigenous communities” – community relationships that “must be continuously ‘relational’ rather than ‘transactional’” (Fawcett et al. 2022:77 − 8).

It is the historically embedded transactional basis of ocean economies – and in particular Western ocean histories – that has overridden ways and means of ocean engagement that might not have capitalist dynamics, and privatisation, at their core. Accordingly, not only does history help us to see ocean privatisation today as part of a longer, variegated, non-linear trajectory, it also helps us to position privatisation in relation to specific modes of oceanic organisation and line drawing (Lambach 2021) as well as law making (Braverman et al. 2023) that violently erase modes of ocean knowledge that rest on other imaginaries of how to live with, and use, the seas without recourse to it being purely resource.

Thinking critically about ocean privatisation can help bring imaginaries – and lived ocean realities beyond the Western canon and realm of experience – to the surface by drawing attention to how they have been suppressed, resisted, and refused, but also by fostering discussion of how they can be reinstated through continued struggle (Satizábal et al. 2024) To illustrate this, we conclude by returning to the example with which we began this article: deep seabed mining. In one sense, a turn to deep seabed mining might seem odd when aiming to illustrate Indigenous, non-capitalist views on the ocean that reject enclosure and allocation. After all, prior to the 20th century no Indigenous human being had directly encountered the deep seabed, let alone mined there. However, the seabed was equally unexperienced by Western humans, and yet few question the legitimacy of their project, over the past fifty years, of drawing on a range of Western legal traditions – from mining law to ocean governance institutions to regulations that prevailed in the near-shore seabed for mining and petroleum extraction – to develop a series of laws for both national continental shelves and The Area (Conde et al. 2022). Given the lack of *all* peoples’ history with the deep seabed, it would seem that Indigenous understandings and legal codes, and ocean engagement practices that exist outside the Western extractivist tradition, are important, legitimate, useful sources for legal inspiration. These might range from belief systems that existed prior to the extractive era (e.g. Childs’ (2020) work on Duke of York Islanders’ seabed cosmologies), to perspectives that joins Indigenous and contemporary scientific understandings of oceanic interconnectivities (e.g. Inuit Circumpolar Council Chair Dalee Sambo Dorough’s perspective on Arctic Ocean seabed allocation (Henriques 2020)). The fact that Indigenous perspectives have barely been considered in contemporary debates over seabed mining regulations speaks, of course, to the power of Western and statist legal norms (Tilot et al. 2021). But it also speaks to the power that understandings of ocean privatisation have in shaping how all of us – proponents and opponents of privatisation – all too often view changes in ocean governance. Perspectives are out there, and have long been, but they remain violently silenced by the hegemony of privatisation discourse – as well as action. To even start to make room for these perspectives in contemporary ocean governance debates – alongside the existent powerful struggles of ocean peoples – it is imperative to question the tendencies that negate privatisation’s complexity and stabilise its history, framing the outer limits of critical debate and marking it off as already settled.

## Footnotes

1 Although we focus here on the US management of the North Pacific pollock fishery, similar points could be made about efforts in other fisheries to institute TACs and ITQs (see, for instance, Fiona McCormack’s (2017) comparative work on, among others, the emblematic cases of New Zealand and Iceland).

2 Data here are derived from twelve months of field work in Seychelles undertaken by the first author of this paper.

3 McDowell et al. (2008) for more on parallels between the spatial imaginaries of the shipping and telecommunications industries.

4 This is not to say that acknowledging other ways of knowing, in and of itself, is enough. All too often, when contemporary ocean governance regimes acknowledge Indigenous knowledge, that knowledge is aimed to be enrolled into existing systems, rather than recognising that the existing system itself is the problem that may be killing the oceans (see Braverman et al. 2023).
